# Patient-reported experiences with side effects of kidney cancer therapies and corresponding information flow

**DOI:** 10.1186/s41687-022-00533-z

**Published:** 2022-12-16

**Authors:** Karin Kastrati, Viktoria Mathies, Anna P. Kipp, Jutta Huebner

**Affiliations:** 1 Klinik Für Innere Medizin II, University Hospital Jena, Am Klinikum 1, 07747 Jena, Germany; 2 UniversitätsTumorCentrum Jena, University Hospital Jena, Jena, Germany; 3 Department of Nutritional Physiology, Institute of Nutritional Sciences, Friedrich Schiller University Jena, Jena, Germany

**Keywords:** Kidney cancer, Nutrition related symptoms, Patient-reported, Renal cell carcinoma, Side effect

## Abstract

**Background:**

Treatment options for metastatic renal cell carcinoma (mRCC) have improved over recent years. Various therapies for metastatic renal cell carcinoma are currently approved for first and successive lines. Having various treatment options makes it important to reflect how patients experience side effects in the real-world setting. So far, data on the side effects of these treatments have only been collected within clinical trials, and have been mostly assessed by the investigator and not as patient-reported outcomes. Our aim was to determine patient-reported experiences of side effects in the real-world setting and to evaluate the doctor-patient communication regarding side effects.

Data were collected via an anonymous, voluntary online survey given to members of a support group for RCC; the questionnaire was completed by 104 mRCC patients.

**Results:**

89.1% of participants were suffering from side effects of any grade. These appeared to be higher for patients treated with tyrosine kinase inhibitors compared to those treated with immune-checkpoint inhibitors (98.4% vs. 68.4%). However, information on side effects is scarce: 4.0% had never heard anything about them while only 18.8% of participants received detailed information on possible side effects. Although 85.6% of participants reported side effects to their physician, 34.6% did not encounter an improvement. Limitations of the study include the design as an online questionnaire and the small sample, consisting only of members of a support group.

**Conclusions:**

Differences can be seen between patient-reported side effects within our survey and those based on clinical trials. A shift towards more patient-reported outcomes is needed. In addition, patients seeking the advice of their physician on side effects are in need of more—or better—information and support.

## Introduction

In 2020, approximately 431,000 new cases of renal cell carcinoma (RCC) were diagnosed world-wide, with an estimated 179,368 deaths [1]. RCC most commonly occurs in the older population (60 + years) and men are more often affected than women (62.9% male and 37.1% female). Risk factors are smoking, obesity, hypertension, kidney stones, occupational exposure to toxic compounds, long-term dialysis and acquired cystic disease. Unfortunately, RCC is often asymptomatic until later stages, so most people are diagnosed with advanced or metastatic disease (mRCC) [1, 2].

Fortunately, treatment options for mRCC have significantly improved over recent years. Since 2006, various therapies have been approved as first and successive lines. Therapeutics can be categorized into three classes: mammalian target of rapamycin inhibitors (mTOR-inhibitors), tyrosine kinase inhibitors (TKIs) and immune-checkpoint inhibitors (ICIs) and they are either administered alone or in combination [2, 3]. The treatment guidelines for renal cell carcinoma provided by the European Society of Medical Oncology (ESMO) were updated in September 2021 and recommend combination therapies of either ICI + ICI or ICI + TKI as first-line treatment throughout all risk groups [3, 4].

Although combination therapies have shown benefits in regard to progression-free survival and overall survival, clinical trials also suggest that they induce more side effects than monotherapies [5–7]. Within those trials more side effects of grade 3 and higher are reported within the group of patients receiving a TKI + ICI combination. Side effects not only reduce the patients' quality of life but are also associated with early treatment discontinuation [8], which may lead to a worse prognosis. So far, data on the side effects of kidney cancer therapies have only been collected within clinical trials, mostly assessed by the investigator and not as patient-reported outcomes (PROs). However, patients tend to down-play the impact of side effects or do not mention them at all when talking to their physician, due to factors such as lack of time or an attitude of not wanting to bother the physician [9]. It could be assumed that investigator-assessment of side effects might lead to underestimating the true burden. Therefore, our objective was to determine patient-reported experiences with side effects in the real-world setting.

Patients also report that information on side effects and side effect management is scarce. A further aim of our survey was to have a closer look at doctor-patient communication in relation to when and how patients are informed about possible side effects and their management.

### Patients and methods

An anonymous, voluntary online survey was conducted among the members of “Das Lebenshaus e.V.” (House of Life), a patient-driven non-profit support group for patients with RCC. The questionnaire was distributed via email to 1258 kidney cancer patients. Inclusion criteria were limited to patients with metastatic kidney cancer with the ability to understand the questions and the willingness to participate. Data were collected from March to July 2020 via SurveyMonkey. Patients were informed about the anonymity of the data and data protection laws were respected.

The final questionnaire consisted of 17 questions separated into the four following sub-categories:

1. Demographic data: age and sex
2. Status of disease: time of diagnosis, time of occurrence of metastases, location of metastases
3. Current therapy and occurring side effects
4. Experience with given information regarding side effects.

Demographic data were assessed using standard questions regarding age and sex. Time of diagnosis and time of occurrence of metastases were assessed using a calendar tool. For the question regarding the location of metastases, we conducted a search within current literature [10] and listed the most common sites of metastasis in a multiple-choice question. Furthermore, participants were given the opportunity to add their own information in an open field. Regarding the question on side effects, the literature on RCC clinical trials was assessed [5–7] and the most common side effects mentioned were listed for participants to choose from (multiple-choice); there was also an option to add others (other side effects, please specify). To determine whether the participants had received information on side effects we used a 4-point ranking scale from “side effects were not mentioned” to “side effects were explained in detail”. To identify patients’ satisfaction with the information given, yes/no questions were used. The survey was approved by the ethics commission of the University Hospital Jena (Number of the ethical vote: 2020–1657 Bef).

Data from the questionnaires were transferred into IBM SPSS Statistics 27. Results were reported as median, means and standard deviation for quantitative variables and frequencies and percentages for categorical variables. Correlations were tested to compare quantitative variables using the Chi-square and t-test. The tests were considered statistically significant if p < 0.05.

## Results

### Demographic data

Of 139 patients who completed the questionnaire, 104 (74.8%) reported that they had metastatic renal cell carcinoma. Furthermore, 98 of these participants (94.2%) stated that they were currently undergoing treatment. 76 participants were male (73.1%) and 28 were female (26.9%). The median age was 63 and more than one-third of the participants (35.6%) had first been diagnosed with RCC within the last 2–5 years. Further characteristics of the study group are shown in Table 1.

**Table 1:** Characteristics of study group (n = 104)

	n (%)
Gender	104 (100)
Male	76 (73.1)
Female	28 (26.9)
Age	103 (100)
Minimum	35
Maximum	82
Median	63
< 50	8 (7.7)
50–59	29 (28.1)
60–69	36 (34.9)
70 +	30 (29.1)
Years from diagnosis	104 (100)
Current year (2020)	2 (1.9)
Last year (2019)	11 (10.6)
2–5 years (2018–2015)	37 (35.6)
6–10 years (2014–2010)	31 (29.8)
> 10 years	23 (21.1)
Diagnosis of metastatic disease	104 (100)
With RCC diagnosis	24 (23.1)
Within 1 year	31 (29.8)
1–5 years from RCC diagnosis	25 (24.0)
6–10 years from RCC diagnosis	9 (8.7)
> 10 years from RCC diagnosis	15 (14.4)

### Information on current therapy

Ninety-seven participants specified their current treatment. Sixty-four (66.0%) were treated with a TKI followed by 19 (19.6%) on ICI monotherapy and 14 (14.4%) undergoing a combination therapy. Most patients in our survey (46.5%) were on first-line treatment for mRCC. A detailed description of treatments is shown in Table 2.

**Table 2:** Type of treatment (n = 97)

	n (%)
Current type of treatment	97 (100)
TKI	64 (66.0)
ICI	19 (19.6)
ICI + ICI	7 (7.2)
ICI + TKI	3 (3.1)
mTOR + TKI	4 (4.1)
Line of treatment	101 (100)
First-line	47 (46.5)
Second-line	20 (19.8)
Third-line	16 (15.8)
Three +	18 (17.8)
Drugs used	
Axitinib	7 (7.2)
Cabozantinib	18 (18.6)
Avelumab + Axitinib	2 (2.1)
Everolimus + Lenvatinib	4 (4.1)
Nivolumab + Ipilimumab	7 (7.2)
Pemrolizumab + Axitinib	1 (1.0)
Lenvatinib (Kisplyx)	3 (3.1)
Nivolumab (Opdivo)	19 (19.6)
Pazopanib (Votrient)	13 (13.4)
Sorafenib (Nexavar)	1 (1.0)
Sunitinib (Sutent)	22 (22.7)

Although the ICI group was rather small (n = 19), it was clear that the demographics were comparable to the TKI group (n = 64). Gender distribution (73.7% male, 26.3% female vs. 71.9%, 28.1%) and median age (68 vs. 64) were balanced. With regard to time of diagnosis, most patients (55.6%) in the ICI group were diagnosed with metastatic disease 2–5 years ago, compared to 34.4% under TKI treatment. Also, patients under ICI seemed to have more former lines of treatment. Please see Table 3 for further information.

**Table 3:** Comparison of TKI and ICI group

	TKI n (%)	ICI n (%)
Gender	64 (100)	19 (100)
Male	46 (71.9)	14 (73.7)
Female	18 (28.1)	5 (26.3)
Age	63 (100)	19 (100)
Minimum	35	52
Maximum	79	82
Median	64	68
Years from diagnosis of metastatic disease	64 (100)	18 (100)
Current year (2020)	14 (21.9)	1 (5.5)
Last year (2019)	15 (23.4)	1 (5.5)
2–5 years (2018–2015)	22 (34.4)	10 (55.6)
6–10 years (2014–2010)	5 (7.8)	5 (27.9)
> 10 years	8 (12.5)	1 (5.5)
Line of treatment	64 (100)	26 (100)
First-line	37 (57.8)	2 (10.5)
Second-line	10 (15.6)	6 (31.6)
Third-line	6 (9.4)	8 (42.1)
Three +	11 (17.2)	3 (15.8)

### Patients’ experiences with side effects

**Fig. 1:**
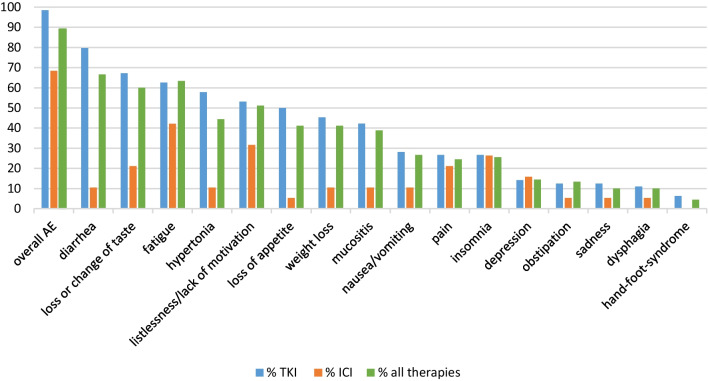
Percentage of side effects according to type of therapy (all: n = 97, TKI: n = 64, ICI n = 19). Blue filled Square % TKI. Orange filled square % ICI. Green filled square % all therapies

In response to the survey, 89.1% of the participants reported that they were suffering from side effects of any grade, with diarrhea being the most common (66.7%) among all forms of therapy, followed by fatigue (63%). Of the 64 participants taking a TKI, 63 reported having side effects of any grade (98.4%) compared to 68.4% in the ICI group (n = 19). Therefore, side effects of any grade were more frequent within the TKI group compared to ICI therapies (98.4% vs. 68.4%; 95%-CI [0.068–0.532]; p = 0.014). Of the participants under TKI therapy, 79.7% reported having diarrhea, compared to 10.5% under ICI treatment. Fatigue occurred in 62.5% of the participants taking a TKI, compared to 42.1% treated with ICI. Half of the patients undergoing TKI treatment suffered from loss of appetite, compared to 5.3% treated with ICI; loss or change of taste was 67.2% versus 21.1%; mucositis 42.2% versus 10.5%; and weight loss occurred in 45.3% compared to 10.5%. Further details on the frequency of side effects in relation to the type of therapy can be seen in Fig. 1.

### Communication of side effects

Hundred and one patients answered the question of whether they had received information on the side effects of the treatment when it was first prescribed. Nineteen (18.8%) stated that they had received detailed information from their physician; 50 patients (49.5%) reported that side effects had been mentioned briefly, 28 participants (27.7%) reported that the physician had talked a little about possible side effects and four participants (4.0%) stated that they had never heard anything at all about possible side effects from their care team.

**Fig. 2:**
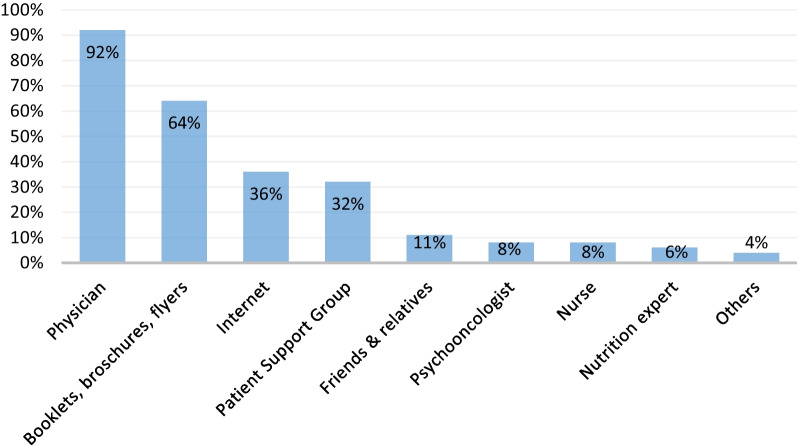
Patients’ source of information about side effects (n = 75)

Eighty-nine patients in our survey (85.6%) reported side effects to their physician; 75 (84.2%) of those said that they received information on how to handle their problems. Within the group of participants who had been well informed, we could see that the most important source of information was the physician, as this was where 92.0% got their information from. This was followed by booklets and information brochures (64.0%), the internet (36%), patient support groups (32%) and friends and relatives (10.6%). Further details on the source of information can be seen in Fig. 2.

Of the 75 participants who had received information on how to handle their side effects after reporting them to the physician, 50 (66.6%) mentioned that the information was easy to understand and that their questions and problems were adequately dealt with. Twenty (26.6%) patients stated that they were well informed but that there was still a need for more support. Overall, 61.3% of the informed patients reported an improvement in side effects after they received the corresponding information. In contrast, 34.6% of the participants in our survey were given information on side effects which did not help to effect an improvement; they were still suffering.

## Discussion

The aim of this survey was to assess patient-reported experiences with side effects of mRCC therapies in the real-world setting and evaluate the doctor-patient communication regarding that topic. The demographic data of the 104 participants included in our survey are typical for other collectives described in studies on mRCC and thus seem to represent the common mRCC patient rather well [1]. Moreover, the data are in line with characteristics reported by the German national registry [11].

Our data show that a vast majority of patients receiving treatment for mRCC (89.1%) is suffering from various degrees of side effects, regardless of the type of therapy. Additionally, it can be seen that TKI therapies seem to cause more adverse effects than ICI treatment (98.4% vs. 68.4%; 95%-CI [0.068–0.532]; p = 0.014). This is consistent with findings in the literature on mRCC treatment [2, 6]. The given combination therapies in our survey were rather varied; therefore, the number of participants taking specific combinations was too low to include those into the analysis. However, clinical trials suggest that especially TKI + ICI combinations have a high burden of side effects [7, 12], which makes it even more important to provide adequate information about side effects and how to deal with them.

In our survey, treatment with TKIs was more often associated with gastrointestinal adverse effects such as diarrhea, loss or change of taste, loss of appetite and weight loss than ICI therapies. This is consistent with reports of adverse reactions observed in clinical trials on mRCC treatment [13, 14]. The results show that almost every second patient encountered weight loss; some of them reported severe loss of weight with e.g. 10 kg in 3 months, 20 kg in 9 months or up to 27 kg within the last year. This is alarming as earlier studies have revealed that malnutrition and weight loss may lead to worsening of treatment efficacy [15]. As most patients have not been accurately informed beforehand about potential gastrointestinal side effects, they do not actively try to maintain their weight and might not be aware of the importance of their nutrition [16]. Although these side effects clearly show a need for nutritional counseling, only 6% of the participants of our survey reported that they had received information from nutrition experts. It appears that there is still a need for more nutritional education and support for patients and an improved awareness among physicians of how important this topic is for the patients’ quality of life as well as for therapeutic efficacy. Nutrition experts such as nutritionists and dieticians should be included in the treatment team and nutritional counseling should start right at the beginning of the therapy.

The results also show a high number of patients suffering from fatigue (63%). This is in line with other studies reporting that fatigue is one of the most common symptoms associated with cancer and cancer treatment [17]. However, it seemed that the number of patients reporting fatigue in our survey was higher than in clinical trials leading to the approval of several TKIs. In these studies, the rate of fatigue ranges from 19% (for tivozanib) to 56% (for cabozantinib), with the other TKIs ranging in between [18–23]. So far, in almost all RCC clinical trials, adverse event severity has been assessed by the investigator using the National Cancer Institute Common Terminology Criteria for Adverse Events. Sometimes questionnaires for health-related quality of life, such as Functional Assessment of Cancer Therapy Kidney Symptom Index (FKSI) questionnaire and the FKSI–Disease-Related Symptoms (FKSI-DRS), have been added. Unfortunately, there has been no assessment of patient-reported outcomes. However, as earlier trials clearly show, reported adverse events can be affected by the methods used for reporting [24–26]. Therefore, there is a great need for including more patient-reported assessments in clinical trials.

In our survey, patients were well aware of the importance of reporting side effects. Nearly 90% mentioned their problems to their physicians. Other studies have reported that direct consultation with the doctor is the primary source of information for cancer patients, followed by other patients, self-help groups and the internet [27, 28]. This is also how the participants in this survey received their information on side effects. Approximately 90% mentioned their physician as being the most important source, followed by booklets and information brochures, the internet or patient support groups. This study clearly shows that even after the consultation with the physician, there is still a need for more or better information and support, which patients try to find by consulting self-help groups or searching the internet. Therefore, it could be helpful if physicians gave out booklets or information brochures for patients to read at home or provided a list of websites containing reliable information.

The majority of participants in our survey was satisfied with the given information on side effects, regardless of where it came from. This is in line with data from other surveys [27, 28]. On one hand, two-thirds of the patients reported that the information they had received regarding the treatment of side effects was well understood and that their questions and problems were adequately dealt with. More than two-thirds could even see an improvement of their side effects. On the other hand, one-third of the participants in our survey did not really understand the information provided and the same number of patients did not encounter any improvement of side effects and therefore continued suffering.

Furthermore, in our survey patients reported that the information on side effects was also scarce when the drugs were first prescribed. Only 19 patients (18.8%) stated that they had received detailed information from their physician; every second patient (49.5%) reported that side effects were mentioned briefly; some (4.0%) stated that they had never heard anything about possible side effects from their care team at all. Therefore, we have to conclude that there is a need for improved doctor-patient communication. When patients are first diagnosed or are given the diagnosis of progression, they are often overwhelmed by the news itself and the amount of information given. Consequently, even if physicians talk about side effects, the patients might not recall those conversations later. Booklets or brochures to hand out to patients could be helpful in that situation. Clearly, more and better information on side effect management should be provided right from the beginning of the therapy. Since living with side effects can seriously impact quality of life, treatment outcomes should not be the only focus of modern therapeutic strategies.

## Limitations

The study design of online research is clearly limited. The return rate was approximately 10%, and it was a relatively small study group (n = 104). Additionally, it seems likely that predominantly those patients who are more familiar with web-based information-seeking returned the online questionnaire. Accordingly, the results cannot be claimed to be representative for all the members of the House of Life or, indeed, all patients with kidney cancer.

It might also be that especially patients with advanced disease and suffering from side effects are contacting support groups. Consequently, they would be more likely to be included in the sample, making it therefore unrepresentative of the wider population of mRCC.

Another limitation is that the survey was undertaken over a period in which the standard of treatment changed from TKI monotherapy to TKI + ICI combinations. Unfortunately, the number of participants in our survey taking combination therapies was too low to compare these results with TKI or ICI monotherapy. Also, the small sample size of 19 in the ICI group might not be representative of this patient population and the conclusions drawn from the comparison with the TKI group might not be reliable. Last but not least, the questionnaire was not validated, although, it is an amalgamation of several instruments which have been used in different patient groups and which have provided consistent results in several other surveys. Unfortunately, the survey did not ask participants to report the severity of their side effects. This could have added more valuable insight and could usefully be the subject of further research.

## Conclusions

Almost all patients who answered the questionnaire in this study were suffering from side effects. Interestingly, there were clear differences between patient-reported side effects within our survey and those given in clinical trials through assessment by investigators. Although this sample is not representative of all kidney cancer patients, this clearly indicates that there needs to be a shift towards more PRO in clinical trials.

Patients seeking the advice of their physician in regards to the treatment of side effects are still in need of more or better information and support. Our results show that there is still a high need for improvement when it comes to doctor-patient communication. Management of side effects should start right from the beginning of the therapy. Furthermore, a high number of patients reported side effects related to gastrointestinal symptoms, such as diarrhea and loss of appetite, which may lead to heavy weight loss, malnutrition and a possible negative impact on treatment outcome. Unfortunately, only a small number of patients had been referred to nutrition experts. Thus, there is obviously a need to increase the nutritional education of patients and improve awareness among physicians. Further research should be conducted to clarify why there is this lack of expert advice when it comes to nutrition-related problems among cancer patients.

## Data Availability

The data that support the findings of this study are available on request from the corresponding author. The data are not publicly available due to privacy or ethical restrictions.
